# Dynamic identifying protein functional modules based on adaptive density modularity in protein-protein interaction networks

**DOI:** 10.1186/1471-2105-16-S12-S5

**Published:** 2015-08-25

**Authors:** Xianjun Shen, Li Yi, Yang Yi, Jincai Yang, Tingting He, Xiaohua Hu

**Affiliations:** 1 School of Computer, Central China Normal University, Wuhan, China; 2 College of Computing and Informatics, Drexel University, Philadelphia, USA

**Keywords:** adaptive density modularity, internal closely associated degree, external closely associated degree, protein functional modules

## Abstract

**Background:**

The identification of protein functional modules would be a great aid in furthering our knowledge of the principles of cellular organization. Most existing algorithms for identifying protein functional modules have a common defect -- once a protein node is assigned to a functional module, there is no chance to move the protein to the other functional modules during the follow-up processes, which lead the erroneous partitioning occurred at previous step to accumulate till to the end.

**Results:**

In this paper, we design a new algorithm ADM (Adaptive Density Modularity) to detect protein functional modules based on adaptive density modularity. In ADM algorithm, according to the comparison between external closely associated degree and internal closely associated degree, the partitioning of a protein-protein interaction network into functional modules always evolves quickly to increase the density modularity of the network. The integration of density modularity into the new algorithm not only overcomes the drawback mentioned above, but also contributes to identifying protein functional modules more effectively.

**Conclusions:**

The experimental result reveals that the performance of ADM algorithm is superior to many state-of-the-art protein functional modules detection techniques in aspect of the accuracy of prediction. Moreover, the identified protein functional modules are statistically significant in terms of "Biological Process" annotated in Gene Ontology, which provides substantial support for revealing the principles of cellular organization.

## Background

As proteins are responsible for driving biological mechanisms and perform physiological functions within the cell [1], investigating the modular structure in protein-protein interaction (PPI) network has been a central content of proteomics studies in the post-genomic era. PPI networks comprising of interconnected protein functional modules dramatically reveals the feature of modular structure -- they have dense connections between the nodes within modules but sparse connections between the nodes in different modules [2]. A module is a fundamental unit formed with highly connected proteins and often possesses specific biological functions [3]. Functional modules can help us to predict the functions of proteins [4]. Accumulated evidences suggest that functional modules are involved in many disease mechanisms [5]. Tracking the functional modules could reveal important insights into modular mechanisms and improve our understanding on the disease pathways etc [6,7]. Though many algorithms to detect protein functional modules have been proposed, yet how to measure the strength of the division of a network into modules (also called communities) has not been explicitly defined. So far the most widely used evaluation criterion for complex networks partitioning is modularity measure Q by Newman and Girvan [8]. However, it has been shown that the Q suffers a resolution limit, that is to say, it performs poorly on identifying small modules [9]. This is mainly because of the global characteristics of the network, which compels the small modules to be concealed in large modules [10]. Using the definition of natural density in number theory, Zhang et al. [11] defined network natural density, which is designed to measure how closely the nodes connecting within communities. Further, he introduced the concept of density modularity, which overcomes the resolution limit in Newman-Girvan algorithm, to evaluate the validity of community partitioning.

Strategies for protein functional modules clustering generally fall into two types - "bottom up" approach proceeding in the form of agglomeration, such as Newman-fast algorithm; and "top down" approach proceeding in the form of division, such as GN algorithm. However, one common drawback to both these two types of approaches is that the protein nodes that have assigned to functional modules have no chance to move into other functional modules during the follow-up processes; instead they are confined to their original modules and result in "module barrier", which lead the erroneous partitioning occurred at previous step to accumulate till to the end.

In this paper, we propose a new algorithm ADM to identify protein functional modules following the introducing of external closely associated degree and internal closely associated degree. In ADM algorithm, a protein opts adaptively whether to stay inside current functional module or move to another module according to the comparison between its internal closely associated degree and external closely associated degree. Owing to the fact that ADM algorithm avoids the shortcoming aforementioned and allows the proteins to dynamically rectify their locations in functional modules whenever necessary, the effectiveness of detecting protein functional modules got improved greatly. The experimental result shows that ADM algorithm outperforms many other state-of-the-art methods such as MCL [12], MCODE [13] and ClusterONE [14] in terms of the accuracy of prediction; moreover, it is capable to identify many protein functional modules with strong biological significance.

## Methods

### Idea of closely associated degree

Functional modules are a cornerstone of many (if not all) biological processes and together they form various types of molecular machineries that perform a vast array of biological functions [15]. So far, the most widely used evaluation criterion for the partitioning of a PPI network into functional modules is global modularity measure Q by Newman and Girvan, the maximum Q corresponds to the optimal partitioning result. However, Q suffers a resolution limit that it cannot effectively identify small clusters, even when these clusters are factions (complete graph). Preliminary observation on considerable amount of PPI networks has indicated that speculating the connections among local protein functional modules through the overall PPI network is the primary cause of the resolution limit.

To overcome the limit of global modularity Q, a new quantitative function (be named density modularity *D*) was introduced to evaluate the validity of community structure partitioning [11]. Density modularity, which represents the degree of tightness inside a functional module, is defined as follows:

(1) $$D=\left[\frac{{l_{i}}^{2}}{{n_{i}}^{2}L}-\frac{{d_{i}}^{4}}{{n_{i}}^{2}L^{3}}\right]$$

Where *L *and *l_i _*denote the number of edges in the entire network and in module *i*, respectively; *d_i _*and *n_i _*denote the sum of the nodes' degree and the number of nodes in module *i*, respectively. The value of *D *ranges from 0 to 1, the same as Q. Density modularity can effectively evaluate the partitioning of a network into communities [11]. We get the optimally partitioning when the density modularity is maximum. In this paper, we introduce the definition of closely associated degree, which represents the increment in density modularity brought by assigning a node to a module. For each protein node, its internal closely associated degree is defined as the closely associated degree between the node and its host module, while its external closely associated degree as the closely associated degree between the node and an external module that connected with it. We calculate the external closely associated degree and internal closely associated degree for each protein node according to the variation of the density modularity during its moving processes. If its external closely associated degree is greater than internal closely associated degree, the node will jump into the corresponding external module; otherwise, it remains in current module.

### Definition of internal closely associated degree

Suppose that the clustering is in progress in a PPI network (the number of its edges is denoted by *L*), *M1 *and *M2 *are the modules to be merged, and the other modules in the network - denoted by *M0 *- remain unchanged. The density modularity corresponding to the entire network is denoted by *D*, and that corresponding to the module *M0 *is denoted by *D0*. Let *l_i_, n_i _*and *d_i _(i = 1, 2) *represent the number of edges, nodes and the sum of the nodes' degree in *Mi*, respectively; the number of the edges between *M1 *and *M2 *is denoted by *e*_12_.

Before the merging of *M1 *and *M2*, we define the density modularity of a PPI network as follows:

(2) $$D=D_{0}+\left[\frac{{l_{1}}^{2}}{{n_{1}}^{2}L}-\frac{{d_{1}}^{4}}{{n_{1}}^{2}L^{3}}\right]+\left[\frac{{l_{2}}^{2}}{{n_{2}}^{2}L}-\frac{{d_{2}}^{4}}{{n_{2}}^{2}L^{3}}\right]$$

After the merging of *M1 *and *M2*, we define the density modularity of the PPI network as follows:

(3) $$D^{\prime }=D_{0}+\left[\frac{{\left(l_{1}+l_{2}+e_{12}\right)}^{2}}{{\left(n_{1}+n_{2}\right)}^{2}L}-\frac{{\left(d_{1}+d_{2}\right)}^{4}}{{\left(n_{1}+n_{2}\right)}^{2}L^{3}}\right]$$

Therefore the variation of density modularity of the PPI network can be formulated as follows:

(4) $$\begin{array}{ccc} \Delta D & =D^{\prime }-D & \\ & =D_{0}+\left[\frac{{\left(l_{1}+l_{2}+e_{12}\right)}^{2}}{{\left(n_{1}+n_{2}\right)}^{2}L}-\frac{{\left(d_{1}+d_{2}\right)}^{4}}{{\left(n_{1}+n_{2}\right)}^{2}L^{3}}\right]-D_{0}-\left[\frac{{l_{1}}^{2}}{{n_{1}}^{2}L}-\frac{{d_{1}}^{4}}{{n_{1}}^{2}L^{3}}\right]-\left[\frac{{l_{2}}^{2}}{{n_{2}}^{2}L}-\frac{{d_{2}}^{4}}{{n_{2}}^{2}L^{3}}\right]\\ & =\frac{1}{L}\left[\frac{{\left(l_{1}+l_{2}+e_{12}\right)}^{2}}{{\left(n_{1}+n_{2}\right)}^{2}}-\frac{{l_{1}}^{2}}{{n_{1}}^{2}}-\frac{{l_{2}}^{2}}{{n_{2}}^{2}}\right]+\frac{1}{L^{3}}\left[\frac{{d_{1}}^{4}}{{n_{1}}^{2}}+\frac{{d_{2}}^{4}}{{n_{2}}^{2}}-\frac{{\left(d_{1}+d_{2}\right)}^{4}}{{\left(n_{1}+n_{2}\right)}^{2}}\right]\\ & \propto \left[\frac{{\left(l_{1}+l_{2}+e_{12}\right)}^{2}}{{\left(n_{1}+n_{2}\right)}^{2}}-\frac{{l_{1}}^{2}}{{n_{1}}^{2}}-\frac{{l_{2}}^{2}}{{n_{2}}^{2}}\right] \end{array}$$

Where both *L *and $\frac{1}{L^{3}}\left[\frac{{d_{1}}^{4}}{{n_{1}}^{2}}+\frac{{d_{2}}^{4}}{{n_{2}}^{2}}-\frac{{\left(d_{1}+d_{2}\right)}^{4}}{{\left(n_{1}+n_{2}\right)}^{2}}\right]$ are constants, which allowed to be omitted during the deviation process of *ΔD*.

Obviously, the variation of density modularity mainly depends on *e*_12_, *l*_1_*+l*_2 _and *n*_1_*+n*_2 _during the merging process of two modules. "Internal Closely Associated Degree" (denoted as *R_in_*^(*s*)^) is defined as the incremental modularity *ΔD *brought when merging a node into its host module. Let *s *represent the module identifier to which the single node originally belongs, then *R_in_*^(*s*) ^can be formulated as follows:

(5) $$\begin{array}{ccc} R_{in}^{\left(s\right)} & =\frac{{\left(l_{1}+l_{2}+{e^{\left(s\right)}}_{in}\right)}^{2}}{{\left(n_{1}+n_{2}\right)}^{2}}-\frac{{l_{1}}^{2}}{{n_{1}}^{2}}-\frac{{l_{2}}^{2}}{{n_{2}}^{2}} & \\ & =\frac{{\left(0+l_{2}+{e^{\left(s\right)}}_{in}\right)}^{2}}{{\left(1+n_{2}\right)}^{2}}-\frac{0^{2}}{1^{2}}-\frac{{l_{2}}^{2}}{{n_{2}}^{2}}\\ & =\;\frac{e^{\left(s\right)}{{}_{in}}^{2}+2le^{\left(s\right)}{}_{in}}{{\left(1+n_{2}\right)}^{2}} \end{array}$$

Where *e*^(*s*)^*_in _*represents the number of edges that connect the node and all the other ones in module *s*.

### Definition of external closely associated degree

"External Closely Associated Degree" (denoted as *R_out_*^(*f*)^) is defined as the incremental modularity *ΔD *brought when merging a node into its adjacent external module. As there often exist more than one adjacent module trying to pull a node out from its original module, the node tends to be merged into the module that offers the greatest closely associated degree. Let *t *mark the module that the node finally selects, then the external closely associated degree can be formulated as follows:

(6) $$\begin{array}{cc} {R_{out}}^{\left(t\right)} & =\underset{t\in \left\{ngbs\right\}}{\text{max}}\left\{\frac{{\left(l_{1}+l_{2}+{e^{\left(t\right)}}_{out}\right)}^{2}}{{\left(n_{1}+n_{2}\right)}^{2}}-\frac{{l_{1}}^{2}}{{n_{1}}^{2}}-\frac{{l_{2}}^{2}}{{n_{2}}^{2}}\right\}\\ & =\underset{t\in \left\{ngbs\right\}}{\text{max}}\left\{\frac{{\left(0+l_{2}+{e^{\left(t\right)}}_{out}\right)}^{2}}{{\left(1+n\right)}^{2}}-\frac{0^{2}}{1^{2}}-\frac{l^{2}}{n^{2}}\right\}\\ & =\underset{t\in \left\{ngbs\right\}}{\text{max}}\left\{\frac{{\left(l_{2}+{e^{\left(t\right)}}_{out}\right)}^{2}}{{\left(1+n_{2}\right)}^{2}}-\frac{{l_{2}}^{2}}{{n_{2}}^{2}}\right\} \end{array}$$

Where *e*^(*f*)^*_out _*is the number of edges connecting the current node and all the nodes within module *t. l*_1 _and *n*_1 _represent the number of edges and nodes when the node is viewed as a single module, respectively. *l*_2 _and *n*_2 _denote the number of edges and nodes in the module to which the node belongs to, respectively. {*ngbs*} represents a collection including all the adjacent external modules that closely associated with the node. Among {*ngbs*}, the node need to find one module that with the greatest closely associated degree to merge into.

Studies show that it is unreasonable to speculate the connections between local protein functional modules through the overall PPI network. In our work, the location of each protein node would be updated constantly according to the comparison between its external closely associated degree and internal closely associated degree. Whether a protein node is to stay inside current module or move to another module, it would contribute to the improving and optimizing of the density modularity of PPI network.

### The overview of ADM algorithm

During the identification of protein functional modules in ADM algorithm, *R_in _*and *R_out _*are directly proportional to the increment of density modularity, which indicates that the nodes' ever move will contributes the greatest increment of density modularity. When merging a protein node into a functional module, if *R_out _*is greater than *R_in_*, the node will move to the corresponding external functional module; otherwise, it will remain inside its original functional module. Therefor the density modularity of a PPI network is variational in the process of detecting functional modules - the value of *R_in _*and *R_out _*for each node need to be recalculated once a node has moved. The nodes in the PPI network will not stop moving until each of them has reached steady state, when the PPI network has been divided into functional modules correctly.

In the initial stage of ADM algorithm, we consider each node as an initial functional module to calculate the closely associated degree between the node and its neighbor modules. Then the node is merged into a neighbor module that with the greatest closely associated degree, which is considered to be the belonging module of the node. When the module structures have reach a stable state, considering all the modules in pairs, we choose a pair to merge if doing this could produce the maximum increment (or minimum reduction) of the sum of density modularity, such a process is repeated until to the end of ADM algorithm. Finally, we take the partition result obtained when the density modularity *D *is maximum as the collection of predicted functional modules.

### ADM algorithm is detailed as follows

(1) Initialize the network as *n *modules, namely, each protein node is took as a separate module.

(2) For each node, the values of its *R_in _*and *R_out _*are calculated respectively, if *R_in_*<*R_out_*, the node moves to the corresponding external functional module; otherwise, it remains inside the original functional module.

(3) Repeat step 2 until all the nodes in PPI network are stable, and record the density modularity *D *when the modules have emerged.

(4) List all the pairs of modules gained from step 3 and suppose each of them has been merged, then we separately calculate the increment of density modularity brought by the merging.

(5) Select a pair of modules that brings to the network the maximum increment (or minimum reduction) of density modularity to merge.

(6) Repeat step 2 to step 5 until the positions of all the nodes remain unchanged.

(7)Pick the partition result obtained when the density modularity *D *gets the maximum value across step 2 to step 6 as the final solution of ADM algorithm.

## Results and discussions

As the yeast PPI network is a relatively credible and complete dataset among the existing PPI networks, it is often used to test the validity of methods for identifying protein functional modules. Among the existing varies versions of yeast PPI network datasets, we choose Gavin dataset [16] and Krogan_extended dataset [17] to compare the performance of ADM algorithm against the following classic clustering algorithms: MCL, MCODE and ClusterONE. Gavin dataset comprising of 7669 interactions among 1855 proteins, and Krogan_extended dataset comprising of 14317 interactions among 3672 proteins, are both removed the self-loop and redundant edges. The known yeast protein functional module set obtained from MIPS contains 236 functional modules, each of which contains at least 3 proteins.

Owing to the randomness inherent to ADM algorithm, it runs 20 times on the above yeast PPI networks and thereby generating 20 groups experimental partition results, among which the one that corresponds to the greatest density modularity is preserved as the final identified functional module set. Each of the identified functional modules contains at least 3 proteins after filtering. As a result, 227 and 442 functional modules are identified, respectively, on Gavin dataset and Krogan_extended dataset. Accuracy metric and GO semantic similarity measurement are employed to evaluate the similarity between the identified protein functional modules and the reference known protein functional modules; besides, we use p-value to evaluate the biological significance of the predicted functional modules.

### Accuracy metric

The harmonic mean of *Sn *(Sensitivity) and *PPV *(Positive Predictive Value), also known as accuracy metric (*Acc*), is typically used to assess the overall performances of varies algorithms.

*Sn *and *PPV *are calculated based on the matching matrix *T *between predicted functional modules and reference functional modules. The number of rows and columns in matrix *T *(denoted as *n *and *m*), represent the number of reference functional modules and predicted functional modules, respectively. The element *t(i,j) *in matrix *T *denotes the number of proteins involved in both the *i*th reference functional module and *j*th predicted protein functional module; and *n(i) *denotes the number of proteins involved in the *i*th reference functional modules. Thus Sn, PPV and Acc can be defined as follows:

(7) $$Sn=\frac{\sum_{i=1}^{n}{\text{max}}_{j=1}^{m}t\left(i,j\right)}{\sum_{i=1}^{n}n\left(i\right)}$$

(8) $$PPV=\frac{\sum_{j=1}^{m}{\text{max}}_{i=1}^{n}t\left(i,j\right)}{\sum_{j=1}^{m}\sum_{i=1}^{n}t\left(i,j\right)}$$

(9) $$Acc=\sqrt{Sn\times PPV}$$

The comparison of ADM algorithm against the following three existing state-of-the-art clustering algorithms is performed by applying them to Gavin dataset and Krogan_extended dataset: ClusterONE (clustering with overlapping neighborhood expansion), MCL (Markov Clustering) and MCODE (Molecular Complex Detection). In terms of accuracy metric, the larger *Sn *to some extent indicates that the more reference protein functional modules could be found, while the lower PPV shows that there exist more predicted protein functional modules that matched with none of reference protein functional modules. As is shown in table 1, *clusters *is the number of identified protein functional modules, *matched *denotes the number of the identified protein functional modules matched with at least one reference functional module. On Gavin dataset, while ADM obtains the second most *clusters *227, it achieves the most *matched *94 in contrast to other algorithms. In addition, ADM obtains the greatest Acc 0.579, which is 15.3% higher than the second best Acc 0.502 achieved by MCL. On Krogan_extended dataset, while ADM obtains the third most *clusters *442, it achieves the most *matched *107 compared with other algorithms. Moreover, the Sn, PPV and Acc obtained by ADM algorithm are 20.5%, 64.2% and 41.7% higher than the second most ones, respectively. In short, ADM algorithm can detect functional modules from PPI network more effectively than all the other three algorithms. By the way, it can be seen that MCODE algorithm performs worst both on these two datasets, which incites us to use only MCL and ClusterONE algorithms for the following comparison.

**Table 1:** Comparative results of varies algorithms on two yeast PPI networks

Dataset	Method	clusters	matched	Sn	PPV	Acc
**Gavin**	ClusterONE	196	82	**0.519**	0.479	0.498
	MCL	**253**	79	0.508	0.497	0.502
	MCODE	135	65	0.426	0.464	0.444
	ADM	227	**94**	0.508	**0.660**	**0.579**

**Krogan_extended**	ClusterONE	**530**	90	0.443	0.402	0.422
	MCL	483	68	0.411	0.408	0.409
	MCODE	64	23	0.199	0.369	0.271
	ADM	442	**107**	**0.534**	**0.670**	**0.598**

### GO semantic similarity measurement

Biologists often compelled to spend much time and a lot of energy on searching biological information, which is attributed to the confusion definitions on biology. Fortunately, Gene ontology (GO) provides a platform to unify the representations of gene and gene product attributes across all species. The ontology covers three domains in terms of cellular component, molecular function and biological process.

GO semantic similarity of a functional module refers to the average associated degree of all the pair-wise proteins within the module [18]. The semantic similarity of cellular component, molecular function and biological process are separately calculated and then the geometric mean of them is took as the functional module's GO semantic similarity. We can obtain the GO semantic similarity by calculating the average weight of all the functional modules. Generally speaking, the greater the GO semantic similarity is, the greater the probability that the proteins perform similar biological functions.

It is convenient for us to calculate the GO semantic similarity of protein functional modules by the tool ProCope [19]. Owing to the poor performance of MCODE algorithm in the above section, here we evaluate the performance of ADM algorithm in terms of GO semantic similarity by comparing it to MCL and ClusterONE algorithms.

As is exhibited in table 2, where MIPS complexes - a collection of protein complexes that has been curated from the biomedical literature - is often used as benchmark for evaluation [20]. On Gavin dataset, despite of the fact that the *Biological Process *achieved by ADM algorithm is lower than that obtained by ClusterONE algorithm, the *Cellular Component *and *Molecular Function *achieved by ADM algorithm, respectively, are 14.2% and 8.9% higher than that obtained by ClusterONE algorithm which has the second best performance here. On Krogan_extended dataset, the *Cellular Component *and *Molecular Function *achieved by ADM algorithm, respectively, are 50.6% and 9.9% higher than that achieved by ClusterONE which also has the second best performance here. Therefore, we have reason to conclude that ADM algorithm not only can identify significant protein functional modules from PPI network but also has better performance than the other algorithms.

**Table 2:** The comparison of varies algorithms on GO semantic similarity

Dataset	Method	Biological Process	Cellular Component	Molecular Function
**Gavin**	ClusterONE	**0.913**	0.769	0.638
	MCL	0.668	0.591	0.494
	ADM	0.748	**0.878**	**0.695**

**Krogan_extended**	ClusterONE	**0.667**	0.508	0.505
	MCL	0.493	0.349	0.397
	ADM	0.586	**0.765**	**0.555**

**MIPS complexes**		0.995	0.921	0.897

### Analysis of P-values

To evaluate the statistical significance of the identified functional modules, many researchers annotate their mainly biological functions by using p-values [21]. Given a predicted functional module with *C *proteins, p-value denotes the probability of observing k or more proteins from the functional module by chance in a biological function shared by *F *proteins from a total genome size of *N *proteins. P-values is formulated as follows:

(10) $$P-value=1-\overset{k-1}{\underset{i=0}{\sum }}\frac{\left(\begin{array}{c} F\\ i \end{array}\right)\left(\begin{array}{c} N-F\\ C-i \end{array}\right)}{\left(\begin{array}{c} N\\ C \end{array}\right)}$$

P-value measures the enrichment degree of a certain function by a protein functional module. The smaller the p-value is, the lower the probability that a biological function arises by chance in the predicted functional module, thus the more significant the predicted functional module is. Given that proteins within a protein functional module are assembled to perform common biological functions, they are expected to share common functions, among which we take the function that corresponding to the minimum p-value as its annotation function. More importantly, the unknown proteins' functions could be predicted according to its belonging functional modules' functions.

Here, we calculate the p-values of *Biological Process *by GO::TermFinder for each identified protein functional modules. GO::TermFinder takes a list of genes as input, and determines whether there are enriched GO terms for that list by searching the shared GO terms or their parents [18]. In table 3, we list some shared GO terms in terms of Gene Ontology term, *Cluster frequency *represents the ratio of the number of proteins that with the corresponding annotations to the total number of proteins in the module.

**Table 3:** The P-values of some functional modules identified by ADM algorithm

Dataset	ID	P-value	Cluster frequency	Gene Ontology term
	1	2.28E-63	40 out of 62 genes, 64.5%	ribosomal large subunit biogenesis
	
**Gavin**	2	6.73E-40	30 out of 46 genes, 65.2%	mitochondrial translation
	
	3	1.58E-37	16 out of 28 genes, 57.1%	tRNA transcription from RNA polymerase III promoter
	
	4	2.03E-35	26 out of 38 genes, 68.4%	mitochondrial translation
	
	5	2.42 E -33	14 out of 22 genes, 63.6%	nuclear-transcribed mRNA catabolic process, exonucleolytic, 3'-5'
	
	6	4.45 E -32	12 out of 15 genes, 80.0%	proteasomal ubiquitin-independent protein catabolic process
	
	7	4.89 E -32	14 out of 21 genes, 66.7%	mRNA polyadenylation
	
	8	1.43 E -31	20 out of 24 genes, 83.3%	mRNA splicing, via spliceosome
	
	9	1.23 E -28	25 out of 25 genes, 100.0%	transcription from RNA polymerase II promoter
	
	10	3.91 E -28	22 out of 24 genes, 91.7%	mRNA metabolic process

**Krogan_extended**	1	2.87 E -43	30 out of 40 genes, 75.0%	mitochondrial translation
	
	2	6.13 E -40	17 out of 28 genes, 60.7%	chromatin disassembly
	
	3	5.52 E -32	23 out of 36 genes, 63.9%	mRNA splicing, via spliceosome
	
	4	1.68 E -26	14 out of 32 genes, 43.8%	rRNA catabolic process
	
	5	2.81 E -26	16 out of 31 genes, 51.6%	histone acetylation
	
	6	3.38 E -23	40 out of 66 genes, 60.6%	transcription, DNA-dependent
	
	7	3.82 E -23	40 out of 66 genes, 60.6%	RNA biosynthetic process
	
	8	1.24 E -21	11 out of 25 genes, 44.0%	mRNA polyadenylation
	
	9	1.31 E -21	22 out of 35 genes, 62.9%	mRNA metabolic process
	
	10	1.18 E -18	9 out of 20 genes, 45.0%	negative regulation of chromatin silencing at telomere

In most situations, the functional module that with p-value<0.01 is considered to be significant. The p-values of most protein functional modules identified by ADM algorithm are lower than 0.01, which indicates the occurrence of these predicted modules does not happen merely by chance. As is exhibited in table 3, the minimum p-value is 2.28E-63, explaining that our algorithm is capable to detect the functional modules with biological significance effectively.

Table 4 lists some examples of functional modules that detected by applying ADM algorithm to Gavin dataset and Krogan_extended dataset. ADM algorithm is capable to detect many large functional modules both in Gavin dataset and Krogan_extended dataset. As is shown in table 4, a functional module that consists of 25 proteins is discovered in Gavin dataset, its clustering frequency is 100%, namely match perfectly, which shows that it enjoys significant biological significance and is probably a real protein functional module. In summary, our ADM algorithm is capable to detect many large functional modules with strong biological significance.

**Table 4:** Examples of functional modules identified from Gavin dataset and Krogan_extended dataset by ADM algorithm

Data set	P-value	Cluster frequency	Gene Ontology term	Predicted functional modules
**Gavin**	1.23e-28	25 out of 25 genes, **100.0%**	transcription from RNA polymerase II promoter	ybr193c ybr253w ycr081w ydr308c ydr443c yer022w ygl025c ygl127c ygl151w ygr104c yhr041c yhr058c ykr095w ylr071c yml007w ymr112c ynl025c ynl236w ynr010w yol051w yol135c yor174w ypl042c ypr070w ypr168w
	
	3.91e-28	22 out of 24 genes, 91.7%	mRNA metabolic process	ybl026w ybr055c ybr152w ycr077c ydl098c ydl160c ydr037w ydr378c ydr473c yer112w yer146w ygl173c ygr075c ygr091w yjl124c yjr022w ylr438c-a ymr080c ymr268c ynl147w ynl256w yor308c ypr082c ypr178w
	
	1.43e-31	20 out of 24 genes, 83.3%	mRNA splicing, via spliceosome	ybr119w ydl087c ydr235w ydr240c yer029c yfl017w-a ygl049c ygr013w ygr074w yhr086w yil061c yjr084w ykl012w ykl204w ylr147c ylr275w ylr298c yml046w ymr125w yol139c yor276w ypl178w ypr057w ypr182w

**Krogan_extended**	2.87e-43	30 out of 40 genes, 75.0%	mitochondrial translation	q0140 yal041w ybl090w ybr006w ybr146w ybr251w ydl045w-a ydr041w ydr124w ydr175c ydr337w ydr347w ydr494w yer050c yer073w ygl129c ygr165w ygr215w yhl004w yhr059w yil070c yil093c yjl063c yjr060w yjr101w yjr113c ykl003c ykl151c ykl155c ymr158w ymr188c ynl081c ynl137c ynl306w ynr036c ynr037c yol143c yor158w ypl013c ypl118w
	
	5.52e-32	23 out of 36 genes, 63.9%	mRNA splicing, via spliceosome	ybr119w ydl087c ydr020c ydr122w ydr235w ydr240c ydr243c ydr247w ydr515w yer029c yfl018w-a ygr013w ygr074w yhr086w yhr165c yil061c yir009w yjl188c ykl012w ykl095w ykr019c ylr147c ylr275w ylr298c ylr318w yml046w ymr001c ymr132c ymr157c yor036w yor159c yor305w yor359w ypl213w ypr057w ypr182w
	
	1.31e-21	22 out of 35 genes, 62.9%	mRNA metabolic process	ybl026w ybl098w ybr055c ycr077c ydl085c-a ydl121c ydl160c ydr055w ydr378c ydr473c yel015w yer112w yer146w yer172c ygl068w ygl173c ygr091w yhr019c yhr140w yjl013c yjl035c yjl124c yjr022w ykl173w ylr419w ylr438c-a ymr268c ynl092w ynl118c ynl147w ynl240c ynr011c yor308c ypr058w ypr178w

## Conclusions

Protein functional module is a fundamental unit formed with highly connected proteins and often possesses specific biological functions [3]. While many algorithms have been developed to detect functional modules, they have a common drawback in terms of "module barrier". In this paper, after thoroughly analyzing the changes in density modularity during the merging process, first we defined the concepts of external closely associated degree and internal closely associated degree, then we proposed a new algorithm to identify protein functional modules based on adaptive density modularity. In ADM algorithm, the partitioning of a PPI network into functional modules always evolves quickly to increase the density modularity of the PPI network, thus ADM algorithm is capable to detect protein functional modules dynamically. Owing to the incorporation of density modularity into the new algorithm ADM, we successfully surmounted the defect of "module barrier" existed in most previously proposed algorithms; moreover, the prediction of protein functional modules got dramatically improved compared with many state-of-the-art algorithms. Therefore, it has important implications for the detection of protein functional modules and the understanding of the principles of cellular organization.

## Competing interests

The authors declare that they have no competing interests.

## Authors' contributions

XS designed the algorithm to identify protein functional modules based on adaptive density modularity. YY implemented the protein functional modules mining algorithm and run the experiments. LY and JY helped plan the experimental analysis and contributed to writing the manuscript. TH and XTH supervised and helped conceive the study. All authors read and approved the final manuscript.

## Funding

This research and publication is supported by the Self-determined Research Funds of CCNU from the Colleges' Basic Research and Operation of MOE (No. CCNU14A02008, CCNU15ZD003), the International Cooperation Project of Hubei Province (No. 2014BHE0017) and the National Natural Science Foundation of China (No. 61170305).

## Notes

This article has been published as part of *BMC Bioinformatics *Volume 16 Supplement 12, 2015: Selected articles from the IEE International Conference on Bioinformatics and Biomedicine (BIBM 2014): Bioinformatics. The full contents of the supplement are available online at http://www.biomedcentral.com/bmcbioinformatics/supplements/16/S12.
